# Application of Non-Destructive Methods: Biomarker Assays in Blood of White Stork (*Ciconia ciconia*) Nestlings

**DOI:** 10.3390/ani11082341

**Published:** 2021-08-08

**Authors:** Dora Bjedov, Alma Mikuška, Carina Lackmann, Lidija Begović, Tibor Mikuška, Mirna Velki

**Affiliations:** 1 Department of Biology, Josip Juraj Strossmayer University of Osijek, 31000 Osijek, Croatia; dora.bjedov@gmail.com (D.B.); amikuska@biologija.unios.hr (A.M.); lbegovic@biologija.unios.hr (L.B.); 2 Department of Evolutionary Ecology and Environmental Toxicology, Goethe University Frankfurt, 60438 Frankfurt am Main, Germany; lackmann@bio.uni-frankfurt.de; 3 Department of Ecosystem Analysis, Institute for Environmental Research, ABBt-Aachen Biology and Biotechnology, RWTH Aachen University, 52074 Aachen, Germany; 4 Croatian Society for Birds and Nature Protection, 31000 Osijek, Croatia; tibor.kopacki.rit@gmail.com

**Keywords:** non-destructive sampling, apex bird species, plasma, S9, biomarkers

## Abstract

**Simple summary:**

Protocols were adjusted and established for biomarker assessment in two blood fractions: plasma and the post-mitochondrial fraction (S9). Basal biomarker values were determined in white stork (*Ciconia ciconia*) nestlings from Croatia, and biomarker responses in two different types of blood samples were compared. Novel fluorescence-based glutathione and reactive oxygen species detection was established providing the potential usage of blood for assessment of environmental impact at the molecular level.

**Abstract:**

White stork (*Ciconia ciconia*) nestlings can provide quantitative information on the quality of the surrounding environment by indicating the presence of pollutants, as they depend on locally foraged food. This study represents the first comparison of biomarkers in two fractions of white stork nestling blood: plasma and S9 (the post-mitochondrial fraction). The aim of this study was to evaluate acetylcholinesterase (AChE), carboxylesterase (CES), glutathione S-transferase (GST), and glutathione reductase (GR), as well as to establish a novel fluorescence-based method for glutathione (GSH) and reactive oxygen species (ROS) detection in plasma and S9. Considering the enzymatic biomarkers, lower variability in plasma was detected only for AChE, as CES, GST, and GR had lower variability in S9. Enzyme activity was higher in plasma for AChE, CES, and GST, while GR had higher activity in S9. Regarding the fluorescence-based method, lower variability was detected in plasma for GSH and ROS, although higher GSH detection was reported in S9, and higher ROS was detected in plasma. The present study indicated valuable differences by successfully establishing protocols for biomarker measurement in plasma and S9 based on variability, enzyme activity, and fluorescence. For a better understanding of the environmental effects on nestlings’ physiological condition, biomarkers can be measured in plasma and S9.

## 1. Introduction

The white stork (*Ciconia ciconia*) is a large migratory bird species breeding in Europe and wintering in Africa, associated with open wet grasslands and agriculture habitats [1]. However, in recent years, studies show that a high percentage of white storks also stay in south-western Europe during winter [2,3]. As an apex bird species with opportunistic feeding habits, their diet mostly comprises of various invertebrates (grasshopper, beetles, earthworms, and crustaceans), amphibians, fish, snakes, lizards, small mammals (voles, mice, rats, and shrews), and, occasionally, trash from landfills [4,5,6,7,8,9]. White stork nestlings are fed on local food sources, foraged by their parents, making them suitable bioindicators and sentinels of contaminants in a local environment [10,11]. A decline in the breeding population of storks is related to decreasing availability of grasslands and wetlands and increase in anthropogenic activities, especially intensive agriculture [12].

Various chemicals are used in agriculture with potential accumulation of toxins in apex bird predators, such as the white stork [12]. Apex bird species are non-target organisms to pesticide exposure in environments such as wetlands or agricultural ponds [13]. Their health status is influenced by their dietary habits, and their body content reflects pollutant concentrations in the food [14,15,16]. Nestling physiological condition is the ability to maintain a stable homeostasis. This condition can be affected by local pollution. Changes in the physiological condition can affect behaviour, cell metabolism, neuronal activity, etc., and therefore can provide useful information regarding local pollution and the effect it has on the environment [15]. Biomarker analysis is utilized for evaluation of a pollutant’s impact on non-target avian species as well as for ecological risk assessment [12]. Enzyme activity measurements in blood can provide valuable information regarding environmental impact on wildlife [17,18]. Enzymatic activity can be altered with various stressors and can provide an early warning sign of pollution [13]. Although some studies still use destructive sampling, such as capturing birds in traps and decapitation [19,20,21,22], blood sampling, if done correctly, is a simple, non-destructive method for laboratory analysis [23] and should be utilized over destructive methods. Non-invasive (e.g., collecting shed feathers [24] and collection of addled eggs [25]) and non-destructive (e.g., blood sampling [26,27]) methods should be employed for the purpose of animal welfare, to minimize the environmental impact on birds, thus helping conserve avian biodiversity, especially when working with near threatened and critically endangered species. For optimal assessment, biomarkers in blood often need to be measured in parallel; therefore, it is recommended to draw a maximum amount of blood at once [28]. Various biomarkers can be measurement in blood, such as antioxidant enzymes, mixed-function oxidases, hormones, corticosteroids [17,18], or environmental contaminants (e.g., lead [29,30]). So far, in avian blood, oxidative stress and esterase biomarkers have been analysed for the purpose of assessing organophosphate and carbamate exposure, air and heavy metal pollution indicators, and genotoxic damage [12,31,32,33,34,35].

Avian blood has diverse implementations in ecotoxicology; however, no data is available assessing the esterase and oxidative stress biomarkers in white stork nestlings in two blood fractions. For this purpose, the main goals of the study were to:

1. Optimize protocols for measurement of the following biomarkers in the collected blood samples, as well as adjust for the microplate reader: acetylcholinesterase, carboxylesterase, glutathione S-transferase and glutathione reductase activities, reactive oxygen species and glutathione levels, as well as total protein content.
2. Determine the basal activities of the measured biomarkers in the blood of white stork nestlings from Croatia.
3. Determine the sex of the white stork nestlings from the sampled blood.

Optimization of biomarker protocols and sex determination in white stork nestlings’ blood will be useful for the purpose of obtaining information from small blood volume, and will enable application of these biomarkers in future research, which will improve the ecotoxicological investigations of birds without the need for destructive sampling. Application of biomarker measurement gives insight into the physiological response to stressors in apex predators and will provide information on early warning signs of possible environmental pollution.

## 2. Materials and Methods

### 2.1. Field Procedure and Blood Extraction

Fieldwork was performed during the 2020 breeding season in villages along Drava river in north-eastern Croatia. The area is influenced by industry near Osijek as well as intensive agrochemical use in the surrounding area. Blood samples were taken from 16 nestlings in 7 nests. Protocols for monitoring the white stork population in Croatia [36,37] were used for finding and approaching nests. All nests were accessed with a telescopic crane. Nestlings were captured in their nest, placed in a bag, and lowered onto the ground. Each nestling was put on its back, and its head covered with a cloth to avoid additional stress. The beak was measured for age determination and all nestlings were between 6 and 8 weeks old. All sampling procedures were done between 08:00 a.m. and 12:00 p.m. to avoid heat stress and to avoid disturbing feeding habits. Morphometric measurements were taken (beak measurement to determine the order of the hatching), and blood samples were collected. A sterile 5 mL syringe and 0.8 mm (20 gauge) needle were used to puncture the brachial vein and approximately 4 mL of blood was drawn and transferred to lithium heparin collection tubes. Blood was stored under cold and dark conditions until centrifugation within 6–8 h. The study was conducted under the permit of The Ministry of Environment and Energy of the Republic of Croatia (Classification code: UP/I-612-07/20-48/130; Registry number: 517-05-1-1-20-4).

### 2.2. Sample Preparation

Blood was centrifuged at 3000× *g* for 10 min at 4 °C. The supernatant (plasma) was transferred to the new sterile tube and kept at –80 °C until further analysis. The pellet was dissolved with a 5 mL 0.1 M phosphate buffer (pH 7.2) and a sonicator was used for cell disruption at 30% strength for 2 min. Samples were subsequently centrifuged at 9000× *g* for 20 min at 4 °C to obtain the post-mitochondrial supernatant (S9). The S9 fraction was kept at –80 °C until further analysis. All measurements were performed in both types of samples: plasma and S9.

### 2.3. Chemicals

In the present study, the following chemicals (analytical grade) were used: acetonitrile (C_2_H_3_N, CAS 75-05-8, 41.053 g mol^−1^), β-Nicotinamide adenine dinucleotide 2′-phosphate reduced tetrasodium salt hydrate (β-NADPH) (C_21_H_26_N_7_Na_4_O_17_P_3_ x H_2_O, CAS 2646-71-1 (anhydrous), 833.35 g mol^−1^ (anhydrous basis)), CellTracker™ Green CMFDA Dye (C_25_H_17_ClO_7_, CAS 136832-63-8, 464.86 g mol^−1^) (ThermoFisher Scientific, Waltham, MA, USA), 1-chloro-2,4-dinitrobenzene (CDNB) (C_6_H_3_ClN_2_O_4_, CAS 97-00-7, 202.55 g mol^−1^), CM-H_2_DCFDA (C_27_H_19_Cl_3_O_8_, CAS 1219794-09-8, 577.8013 g mol^−1^) (ThermoFisher Scientific, Waltham, MA, USA), (2-Mercaptoethyl) trimethylammonium iodide acetate (acetylthiocholine iodide) (CH_3_COSCH_2_CH_2_N(CH_3_)_3_I, CAS 1866-15-5, 289.18 g mol^−1^), disodium hydrogen phosphate (NaH_2_PO_4_, CAS 7558-79-4, 141.957 g mol^−1^), 5,5′-dithiobis-(2-nitrobenzoic acid) (DTNB) ([-SC_6_H_3_(NO_2_)CO_2_H]_2_, CAS 69-78-3, 396.35 g mol ^−1^), glutathione disulfide (GSSG, C_20_H_32_N_6_O_12_S_2_, CAS 27025-41-8, 612.6 g mol^−1^), *p*-nitrophenyl acetate (C_8_H_7_NO_4_, CAS 830-03-5, 181.147 g mol^−1^), (2*S*)-2-amino-4-{[(1*R*)-1-[(carboxymethyl)carbamoyl]-2-sulfanylethyl]carbamoyl}butanoic acid (glutathione (GSH)) (C_10_H_17_N_3_O_6_S,CAS 70-18-8, 307.32 g mol^−1^), and sodium dihydrogen phosphate dihydrate (NaH_2_PO_4_ × 2H_2_O, CAS 13472-35-0, 156.006 g mol^−1^). For protein concentration measurements, the Pierce™ BCA Protein Assay Kit (Pierce Biotechnology, Waltham, MA, USA) was used.

### 2.4. Enzymatic Biomarkers

All biomarker measurements were adjusted for the Tecan Spark 10 M microplate reader (Tecan Trading AG, Männedorf, Switzerland). The plasma and S9 samples as well as blanks were measured in triplicate. Enzyme activity was calculated from the obtained changes in the measured absorbance and expressed as specific enzyme activity.

#### 2.4.1. Protocol for Measurement of Acetylcholinesterase (AChE) Activity

The activity of AChE in the plasma and S9 samples was determined according to the method of Ellman et al. [38]. For the plasma samples, the reaction mixture contained 5 µL plasma diluted 5x with phosphate buffer (0.1 M, pH 7.2), 180 µL phosphate buffer (0.1 M, pH 7.2), 10 µL DTNB (1.6 mM, prepared with phosphate buffer (0.1 M, pH 7.2)), and 10 µL acetylthiocholine iodide (156 mM, prepared with distilled water). Increase in absorbance was measured for 5 min at 412 nm. For the S9 samples, the reaction mixture contained 25 µL S9 diluted 10x with phosphate buffer (0.1 M, pH 7.2), 180 µL phosphate buffer (0.1 M, pH 7.2), 10 µL DTNB (1.6 mM prepared with phosphate buffer (0.1 M, pH 7.2)), and 10 µL acetylthiocholine iodide (156 mM, prepared with distilled water). Increase in absorbance was measured for 10 min at 412 nm. Blank measurements of the plasma and S9 were performed in parallel containing 180 µL phosphate buffer, 10 µL DTNB, and 10 µL acetylthiocholine iodide (all prepared in the same way as described previously). Specific enzyme activity was calculated with the extinction coefficient (ε) = 13.6 × 10^3^ M^−1^ cm^−1^.

#### 2.4.2. Protocol for Measurement of Carboxylesterase (CES) Activity

The activity of carboxylesterase in plasma and S9 was determined according to the Hosokawa and Satoh method [39]. For the plasma samples, the reaction mixture contained 10 µL plasma and 150 µL *p*-nitrophenyl acetate (1 mM, dissolved in acetonitrile, diluted with distilled water). Increase in absorbance was measured for 4 min at 405 nm. For the S9 samples, the reaction mixture contained 20 µL S9 10x diluted with phosphate buffer (0.1 M, pH 7.2) and 150 µL *p*-nitrophenyl acetate (1 mM, prepared in acetonitrile, diluted with distilled water). Blank measurements of the plasma and S9 were performed in parallel containing 150 µL *p*-nitrophenyl acetate (prepared in the same way as described previously). Increase in absorbance was measured for 5 min at 405 nm. Specific enzyme activity was calculated with ε = 16.4 × 10^3^ M^−1^ cm^−1^.

#### 2.4.3. Protocol for Measurement of Glutathione S-Transferase (GST) Activity

The activity of glutathione S-transferase in plasma and S9 was determined following the Habig and Jakoby method [40]. For the plasma samples, the reaction mixture contained 5 µL plasma, 160 µL CDNB (1 mM, dissolved in 96% ethanol and diluted with phosphate buffer (0.1 M, pH 7.2)), and 40 µL GSH (25 mM, prepared in distilled water). Increase in absorbance was measured for 2 min at 340 nm. For the S9 samples, the reaction mixture contained 20 µL S9 homogenate diluted 10x with phosphate buffer (0.1 M, pH 7.2), 160 µL CDNB (1 mM, dissolved in 96% ethanol and diluted with 0.1 M, pH 7.2 phosphate buffer), and 40 µL GSH (25 mM, prepared in distilled water). Blank measurements of the plasma and S9 were performed in parallel containing 160 µL CDNB and 40 µL GSH (all prepared in the same way as described previously). Increase in absorbance was measured for 5 min at 340 nm. For the plasma measurement, the first minute was needed for stabilization and was omitted from the calculations. Specific enzyme activity was calculated with ε = 9.6 × 10^3^ M^−1^ cm^−1^.

#### 2.4.4. Protocol for Measurement of Glutathione Reductase (GR) Activity

The activity of glutathione reductase in plasma and S9 was determined using the Habig and Jakoby protocol [40]. For the plasma samples, the reaction mixture contained 20 µL plasma, 100 µL phosphate buffer (0.1 M, pH 7.2), 100 µL GSSG (2 mM, prepared in phosphate buffer (0.1 M, pH 7.2)), and 10 µL β–NADPH (1 mM, prepared in phosphate buffer (0.1 M, pH 7.2)). Decrease in absorbance was measured for 10 min at 340 nm. For the S9 samples, the reaction mixture contained 10 µL S9, 100 µL phosphate buffer (0.1 M, pH 7.2), 100 µL GSSG (2 mM, prepared in phosphate buffer (0.1 M, pH 7.2)), and 10 µL reduced β–NADPH (1 mM, prepared in phosphate buffer (0.1 M, pH 7.2)). Blank measurements for plasma and S9 were performed in parallel containing 100 µL phosphate buffer, 100 µL GSSG, and 10 µL reduced β–NADPH (all prepared in the same way as described previously). Decrease in absorbance was measured for 10 min at 340 nm. Specific enzyme activity was calculated with ε = 6.22 × 10^3^ M^−1^ cm^−1^.

### 2.5. Fluorescent Dyes Protocols

Detection of GSH and ROS using the fluorescent dyes was conducted based on the protocol previously developed for zebrafish larvae [41] and adjusted here for avian plasma and S9 samples. Measurements were conducted using the Tecan Spark 10 M microplate reader with the following settings: excitation wavelength—485 nm; emission wavelength—530 nm; and gain—50. Each plasma, S9, blank, and positive control sample was performed in parallel and measured in triplicate.

#### 2.5.1. CellTracker™ Green CMFDA (GSH) Dye

For the plasma samples, the reaction mixture contained 2 µL plasma, 90 µL phosphate buffer (0.1 M, pH 7.2), and 5 µL CellTracker™ Green CMFDA (9.78 µM, prepared in DMSO). Fluorescence was measured every 5 min for 60 min. For the S9 samples, the reaction mixture contained 2 µL S9, 90 µL phosphate buffer (0.1 M, pH 7.2) and 5 µL CellTracker™ Green CMFDA (9.78 µM, prepared in DMSO). Fluorescence was measured every 5 min for 60 min. The blank reaction mixture contained 90 µL phosphate buffer and 5 µL CellTracker™ Green CMFDA (prepared in the same way as described previously) and the positive control reaction mixture contained 2 µL GSH (25 mM, prepared in distilled water), 90 µL phosphate buffer, and 5 µL CellTracker™ Green CMFDA (all prepared in the same way as described previously) for both the plasma and S9 samples. The first 30 min were used for calculations due to the optimal linear increase for plasma and S9.

#### 2.5.2. CM-H_2_DCFDA (ROS) Dye

For plasma samples, the reaction mixture contained 10 µL plasma, 90 µL phosphate buffer (0.1 M, pH 7.2), and 10 µL CM-H_2_DCFDA dye (7.87 µM, prepared in DMSO). Fluorescence was measured every 5 min for 30 min. For the S9 samples, the reaction mixture contained 10 µL S9, 90 µL phosphate buffer (0.1 M, pH 7.2), and 5 µL CM-H_2_DCFDA dye (7.87 µM, prepared in DMSO). Fluorescence was measured every 5 min for 120 min. The blank reaction mixture contained 90 µL phosphate buffer and 5 µL CM-H_2_DCFDA dye (prepared in the same way as described previously), and the positive control reaction mixture contained 2 µL H_2_O_2_ (0.019 M, prepared in distilled water), 90 µL phosphate buffer, and 5 µL CM-H_2_DCFDA dye (prepared the same way as described previously) for both plasma and S9.

### 2.6. Protein Quantification Assay

Protein quantification was performed using the Pierce^TM^ BCA Protein Assay Kit and measurements were performed using the Tecan Spark 10 M microplate reader. The working solution was prepared as described in the protocol provided in the kit, with bovine serum albumin as a standard. Each plasma, S9, blank, and standard sample was performed in parallel and measured in triplicate. For the plasma samples, the reaction mixture contained 2.5 µL diluted plasma (5x with phosphate buffer, 0.1 M, pH 7.2), 22.5 µL phosphate buffer (0.1 M, pH 7.2), and 200 µL working solution. For the S9 samples, the reaction mixture contained 2.5 µL diluted S9 (10x diluted with phosphate buffer, 0.1 M, pH 7.2), 22.5 µL phosphate buffer (0.1 M, pH 7.2), and 200 µL working solution. The microplate with reaction mixture was shaken for 30 s in Tecan Spark 10 M microplate reader, incubated at room temperature for 2 h, and the protein concentration was determined at 562 nm.

### 2.7. Sex Determination

DNA was isolated using an extraction buffer containing 10 mM EDTA, 10 mM Tris-Cl (pH 8.0), 100 mM NaCl, 2% sodium dodecyl sulphate (SDS, Carl Roth GmbH, Karlsruhe, Germany), and ultrapure water in final concentrations. In a sterile tube, 125 µL S9, 360 µL extraction buffer, 10 µL proteinase K (10 mg mL^−1^ stock concentration), and 16 µL 1 M dithiothreitol (DTT, Carl Roth GmbH) were added. Following incubation on a thermo–shaker for 30 min, 56 °C at 1000 rpm, 200 µL 3 M sodium acetate (Carl Roth GmbH) was added, vortexed, and incubated for 5 min on ice. The samples were centrifuged for 10 min at 16,000× *g*, at 4 °C, after which the supernatant was transferred to a new tube. Ice-cold isopropanol was added to the supernatant 1:1 (v:v) for DNA precipitation. The samples were briefly shaken and then incubated for 30 min at –20 °C. Afterwards, the samples were centrifuged at 18,000× *g* for 20 min at 4 °C, the supernatant was discarded, and the pellet was washed with 1 mL 70% ethanol. Samples were centrifuged at 18,000× *g*, at 4 °C, for 90 s and the supernatant was discarded. DNA was air-dried and dissolved in 10 µL of nuclease-free water, vortexed, and centrifuged. For DNA quantification, a NanoPhotometer (Implen GmbH, München, Germany) was used. For the sex-specific *CHD* gene [42], the amplification and visualising PCR products protocol by Begović et al. [43] was followed.

### 2.8. Data Analysis

Data analyses were performed using GraphPad Prism software version 8.4.3 [44]. Normality of the data was confirmed with a Shapiro–Wilk test. To compare the difference between the means of the biomarker response in plasma and S9, Welch’s *t*-test was used as unequal variances were confirmed with the F-test. The level of statistical significance (*p*) was 0.05. Response variability in plasma and S9 for each parameter was calculated by dividing the standard deviation of the obtained data with the mean of the obtained data. All results are expressed as the mean ± SD and presented as bar plots.

## 3. Results and Discussion

### 3.1. Sex Determination

Sex was determined from S9 using the *CHD* gene. Sex-typing showed 8 males and 8 females (Figure S1). There were no statistical differences in biomarker response regarding sex. Various volumes of S9 were used, and the optimal protocol was determined based on DNA quantity and quality, as shown in Table S1. DNA quality was determined from A _260/280_ and A _260/230_, indicating purity [45,46,47]. The average A _260/280_ was 1.99 ± 0.06. A ratio of ≥ 1.8 is accepted and considered uncontaminated DNA [48]. A ratio of ≤ 1.6 may indicate presence of protein, phenols, or other impurities absorbing at 280 nm [49]. The average A _260/230_ was 2.02 ± 0.18. A ratio of 2.00–2.20 is considered uncontaminated DNA. If A _260/230_ is lower, salt, lipid, protein, phenol, guanidinium chloride, or EDTA contamination is suspected [50,51]. If the two samples have the same A _260/280_, but different A_260/230_, this may be due to different sample concentrations [52]. During blood sampling, blood coagulation is possible, decreasing the sample concentration. During the sonication process, there are less available cells, as the samples do not have equal homogeneity; therefore, the DNA yield will be lower. Although coagulated samples cannot be used for enzyme assays, they can be used for DNA analysis, e.g., sex determination or DNA methylation [53].

### 3.2. Enzymatic Biomarkers

#### 3.2.1. Overview of the Results

Results of the enzymatic biomarkers and fluorescent dyes analysed in plasma and S9 of white stork nestlings are presented in Table 1. Enzymatic biomarkers were analysed in either plasma or S9; however, when measuring several parameters in blood, there are certain limitations due to sample volume. Therefore, the enzymatic response in plasma and S9 samples was investigated. In case of a limited sample volume, the results of this study will help in deciding which biomarker should be chosen for measurement in which sample type. Enzymatic biomarkers from blood could be used to identify changes in biomarker response regarding geographical differences, weather conditions, environmental pollution gradient, age differences (nestlings, fledglings, juvenile, and adults), and clutch and brood size. Furthermore, results of the study could be implemented and help in the monitoring of the white stork population health status in the future.

#### 3.2.2. Acetylcholinesterase and Carboxylesterase Activity

An increase in absorbance for acetylcholinesterase (AChE) plasma and S9 (Figure S2) were observed for 5- and 10-min periods, respectively. Different sample concentrations and measurement times were used and determined based on a linear absorbance increase and R^2^ ≥ 0.95. Due to high AChE activity, plasma and S9 were diluted prior to measurement. Plasma samples were diluted 5 times, whereas the S9 samples were diluted 10 times because avian erythrocytes contain haemoglobin that interferes in the absorbance spectrum 400–415 nm. To obtain satisfactory results, the S9 samples had to be more diluted and the measurement times were prolonged, to reduce the haemoglobin influence on the assay, as shown in AChE activity in rat erythrocytes [54].

**Figure 1:**
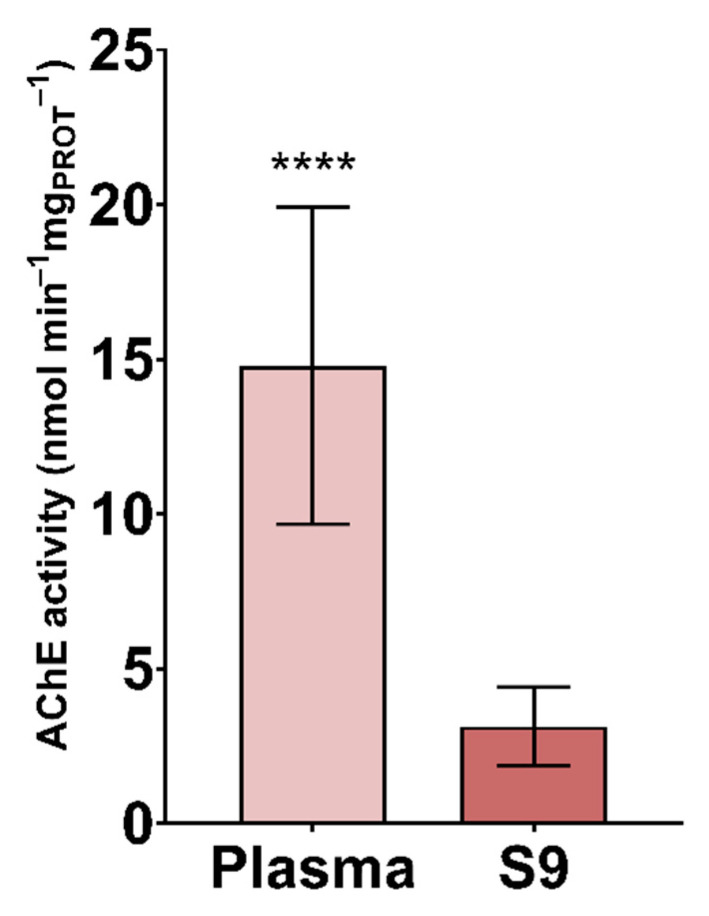
Specific activity of acetylcholinesterase (AChE) in plasma and S9 (nmol min^−1^ mg_PROT_^−1^) of white stork (*C. ciconia*) nestlings (*n* = 16), presented as the mean ± SD. Statistical difference is indicated with **** (Welch’s *t*-test, *p* < 0.0001).

The results of AChE activity in plasma and S9 are shown in Figure 1. Significantly higher specific AChE activity was reported in plasma than in S9 (*p* < 0.0001). However, lower variability among samples was observed in plasma than S9 (Table 1). AChE, as a transmitter hydrolysing acetylcholine, is primarily found in the central and peripheral nervous system as well as muscular system [55]. There is no data available for AChE activity in the blood of white stork nestlings. However, blood AChE histochemistry was assessed [56], and AChE activity was analysed for the purpose of determining the effects of daily photoperiods, a behaviour biomarker of organophosphate (OP) exposure, to establish the basal levels, compare the response to organophosphate and carbamate exposure, and compare the age-dependent changes in plasma [19,20,21,57,58,59,60]. Furthermore, plasma cholinesterases were characterised to establish the basal activities [34,61]. There is wide variation in AChE activity interspecies [57] and between matrices, pointing out the need to determine the basal AChE activity in plasma and S9 in white stork nestlings. Lower AChE activity in S9 may be due to AChE localization—bound to erythrocyte membranes [62,63] that are destroyed with sonication and centrifugation. After S9 preparation, the pellet containing cell membranes is usually discarded.

An increase in carboxylesterase (CES) absorbance (OD) was observed for plasma and S9 samples (Figure S3) for 2- and 5-min time periods, respectively. Different sample concentrations and measurement times were tested, and the final values used are based on a linear absorbance increase and R^2^ ≥ 0.95. In the S9 samples, the measurement time was prolonged due to a high haemoglobin concentration, interfering with the assay [54]. Nevertheless, a linear increase could be observed.

**Figure 2:**
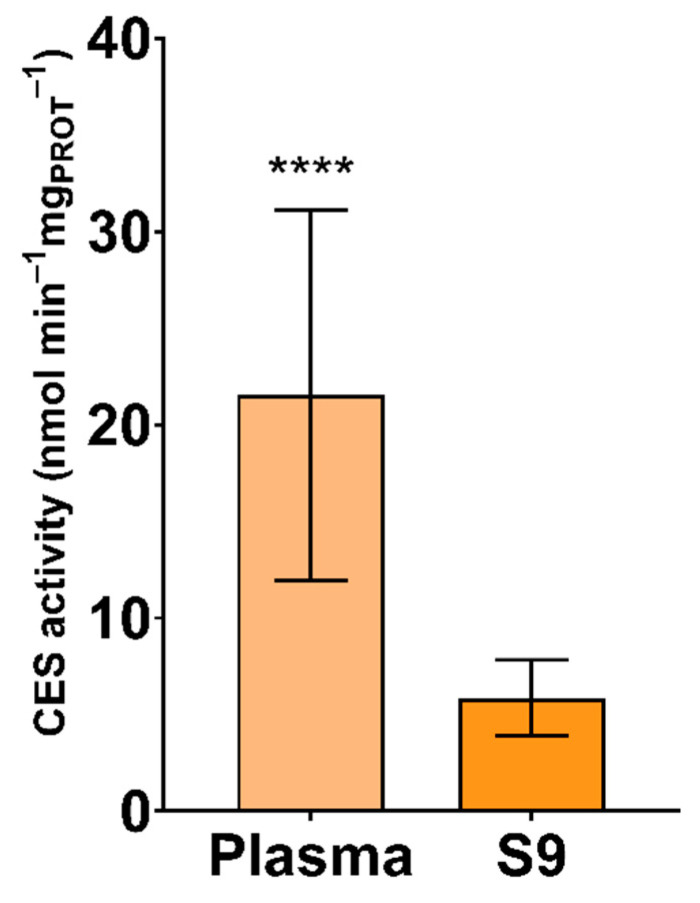
Specific activity of carboxylesterase (CES) in plasma and S9 (nmol min^−1^ mg _PROT_^−1^) of white stork (*C. ciconia*) nestlings (*n* = 16), presented as the mean ± SD. Statistical difference is indicated with **** (Welch’s *t*-test, *p* < 0.0001).

The results of CES activity in plasma and S9 are shown in Figure 2. Significantly higher specific CES activity was reported in plasma than in S9 (*p* < 0.0001). Moreover, higher variability among samples was observed in plasma than S9 (Table 1). CES is a ubiquitous enzyme, with the main function being the hydrolysation of carboxylic acid esters to acid and alcohol, a detoxification mechanism for various xenobiotics [64,65]. CES activity has previously been measured in blood of pigeons (*Columba livia*) and several bird of prey species for the purpose of evaluating CES activity as a potential biomarker of OP exposure [31]. CES and cholinesterase activity was determined in the muscle and liver of yellow-legged gull (*Larus michahellis*) for the purpose of monitoring environmental pollution [66]. Furthermore, CES activity was measured in blood of white storks (*C. ciconia*), black storks (*Ciconia nigra*), vultures, and diurnal and nocturnal predatory birds for the purpose of evaluating CES activity as a potential biomarker of OP and carbamate exposure [67]. Specific CES activity was higher in plasma than in S9, due to low esterase activity in avian erythrocytes [68].

AChE and CES have possible applications in avian species for environmental biomonitoring, as exposure biomarkers to diverse environmental pollutants. AChE is usually regarded as a destructive biomarker, since it is analysed in brain tissue [69], which is not suitable for endangered species, making this non-destructive evaluation essential. Although CES is usually analysed in serum, and therefore is considered a non-destructive biomarker (e.g., [70]), certain limitations exist, e.g., the blood volume that could be taken without harming the bird. Due to esterase’s variability between avian species, it is important to determine the basal activity for each species, as well as to determine activity in plasma and S9.

#### 3.2.3. Glutathione S-Transferase and Glutathione Reductase Activity

The increase in glutathione S-transferase (GST) absorbance (OD) was observed for plasma and S9 samples (Figure S4) for 1- and 5-min time periods, respectively. Different sample concentrations and measurement times were tested, and the final values used are based on a linear absorbance increase and R^2^ ≥ 0.95. For S9, due to haemoglobin interference [54], the measurement was prolonged.

**Figure 3:**
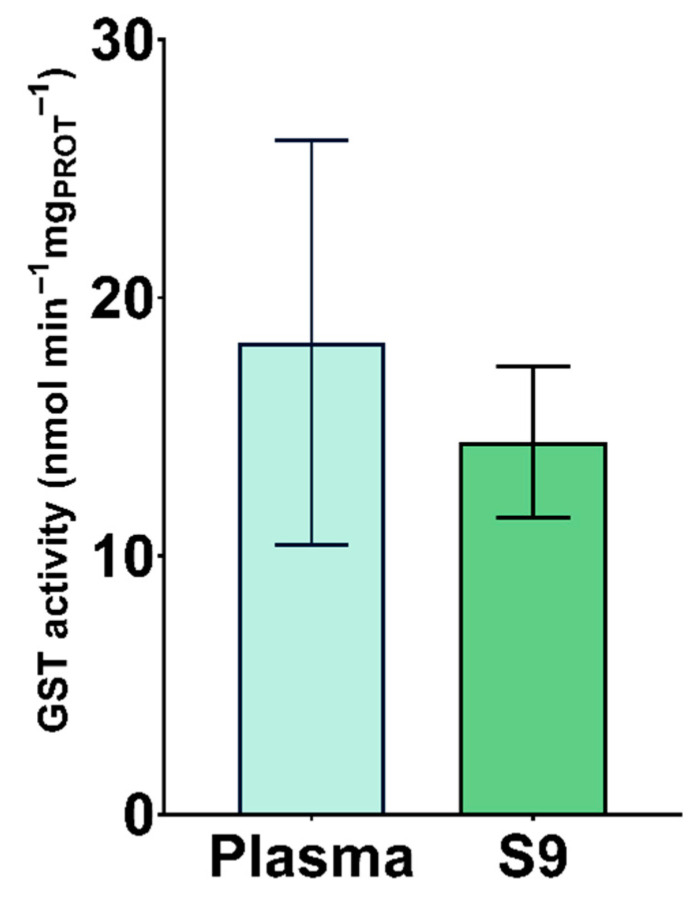
Specific activity of glutathione S-transferase (GST) in plasma and S9 (nmol min^−1^ mg _PROT_^−1^) of white stork (*C. ciconia*) nestlings (*n* = 16), presented as the mean ± SD.

The results of GST activity in plasma and S9 are shown in Figure 3. There was no statistical difference between specific GST activity in plasma and S9, although higher variability among samples was observed in plasma than S9 (Table 1). GST is an enzyme catalysing GSH to xenobiotic substrate conjugates, as a detoxification mechanism [71,72]. As shown in Figure 3, specific GST activity was similar in plasma and S9 of white stork nestlings due to the enzyme distribution in these two blood fractions. Since GST’s primary function is xenobiotic metabolism, it can be found intra- and extracellular [73]. Plasma GST detection and its activity reflects de novo synthesis in the liver [74]. So far, GST has been analysed in the blood of various avian species for the purpose of assessing oxidative stress caused by metal pollution and persistent organic pollutants as well as evaluating physiological conditions due to environmental stress [35,75,76,77,78,79,80,81,82,83,84,85]. When comparing GST activity in S9 between nestling, juvenile, and adult storks, Oropesa et al. [35] reports 877.72 nmol min^−1^ mg_PROT_^−1^ in juveniles, and 964.61 nmol min^−1^ mg_PROT_^−1^ in adults, considerably higher than reported in this study for nestlings.

A decrease in glutathione reductase (GR) absorbance (OD) was observed in plasma and S9 (Figure S5) for the 10-min time period. Different sample concentrations and measurement times were tested, and the final values used are based on a linear absorbance increase and R^2^ ≥ 0.95.

**Figure 4:**
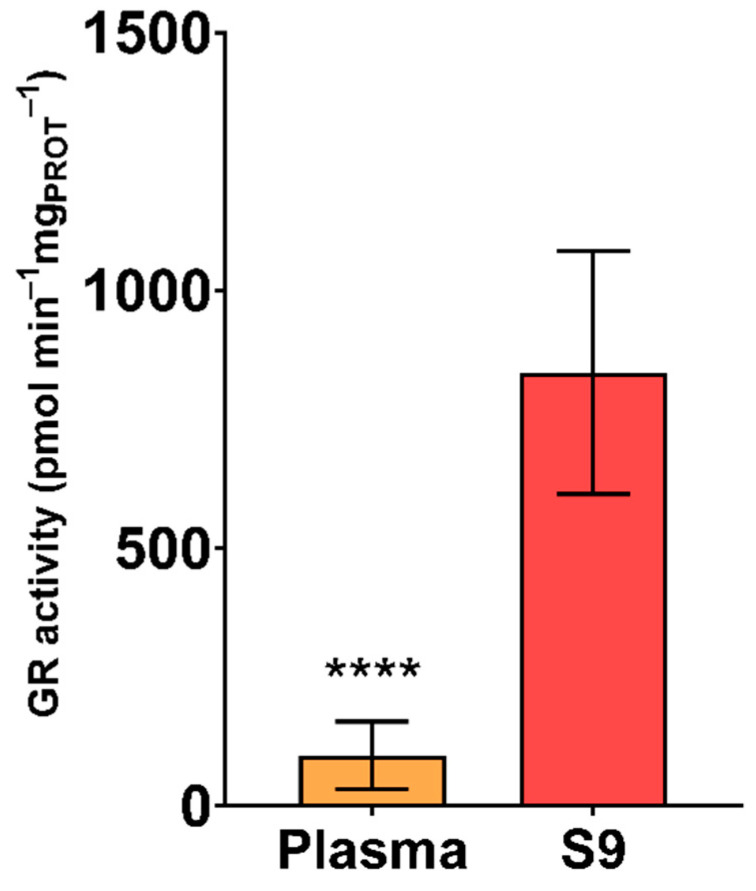
Specific activity of glutathione reductase (GR) in plasma and S9 (pmol min^−1^ mg_PROT_^−1^) of white stork (*C. ciconia*) nestlings (*n* = 16), presented as the mean ± SD. Statistical difference is indicated with **** (Welch’s *t*-test, *p* < 0.0001).

The results of GR activity in plasma and S9 are shown in Figure 4. Significantly higher specific GR activity was reported in S9 than in plasma (*p* < 0.0001). Furthermore, higher variability among samples was observed in plasma samples compared to S9 samples (Table 1). GR is an enzyme catalysing the NADPH-dependent reduction of GSSG to GSH. GSSG reduction is an essential reaction for the preservation of GSH levels, since GSH has a primary function in processes regarding oxidation and reduction, as well as cellular detoxification [86]. GR has been measured in avian blood for the purpose of assessing ecophysiological determination and antioxidant defences as a response to environmental pollution, in addition to evaluating the effect of oxidized fat and selenium on GR activity [35,78,79,83,84,87,88,89,90,91]. Oropesa et al. [35] reports that the GR activity in S9 of juvenile and adult storks (*C. ciconia*) is substantially lower (410 pmol min^−1^ mg_PROT_^−1^ and 380 pmol min^−1^ mg_PROT_^−1^, respectively) than reported in this study for white stork nestlings. This could be due to production of free radicals and depletion of antioxidant defences, both related to aging and age-related diseases [92,93]. Considering that older storks loose function to regulate physiological homeostasis and depletion of some blood enzymatic antioxidants, as a consequence of aging [94,95,96], nestlings might be a more suitable age group for biomonitoring assessments. As shown in Figure 4, higher GR activity was found in S9 than plasma. That being said, GR is a cellular enzyme that accumulates in cellular regions with high electron flux, resulting in high ROS production [97].

Measuring oxidative stress parameters in blood has certain restrictions, e.g., fieldwork limitations and small sample volumes. Our work demonstrates that oxidative stress biomarker measurements could be performed by using either plasma or S9 if there is limitation of the sample volume. When interpreting the results, it is also important to take into account that oxidative stress might not originate in the circulation system but in other tissue; therefore, it is necessary to analyse several biomarkers in different matrices for a broad view of the physiological condition. For this purpose, we evaluated GST and GR in two blood fractions, giving insight into their activity in plasma and S9.

### 3.3. Fluorescent Dyes

The fluorescence-based assay for GSH detection has been successfully established in avian plasma and S9, confirmed by a positive control in which a substrate (GSH) was added resulting in 17 times higher fluorescence detection in the positive control than the blanks for plasma, and 19 times higher fluorescence detection in the positive control than blanks in S9. Furthermore, the fluorescence-based assay for ROS detection was also successfully established in avian plasma and S9, confirmed by a positive control in which a substrate (H_2_O_2_) was added, resulting in 12 times higher fluorescence detection in the positive control than blanks for plasma, and 3 times higher fluorescence detection in the positive control than blanks in S9.

CellTracker^TM^ Green CMFDA dye was used for GSH detection. Different sample concentrations and measurement times were tested, and the final values used are based on a linear fluorescence increase and R^2^ ≥ 0.95 (Figure S6). Increase in fluorescence (RFU) was observed in plasma for 60 min. Fluorescence (RFU) in S9 was measured for 120 min, and an optimal linear increase was observed in the first 30 min, after which GSH saturation was observed, resulting in a stagnation line.

**Figure 5:**
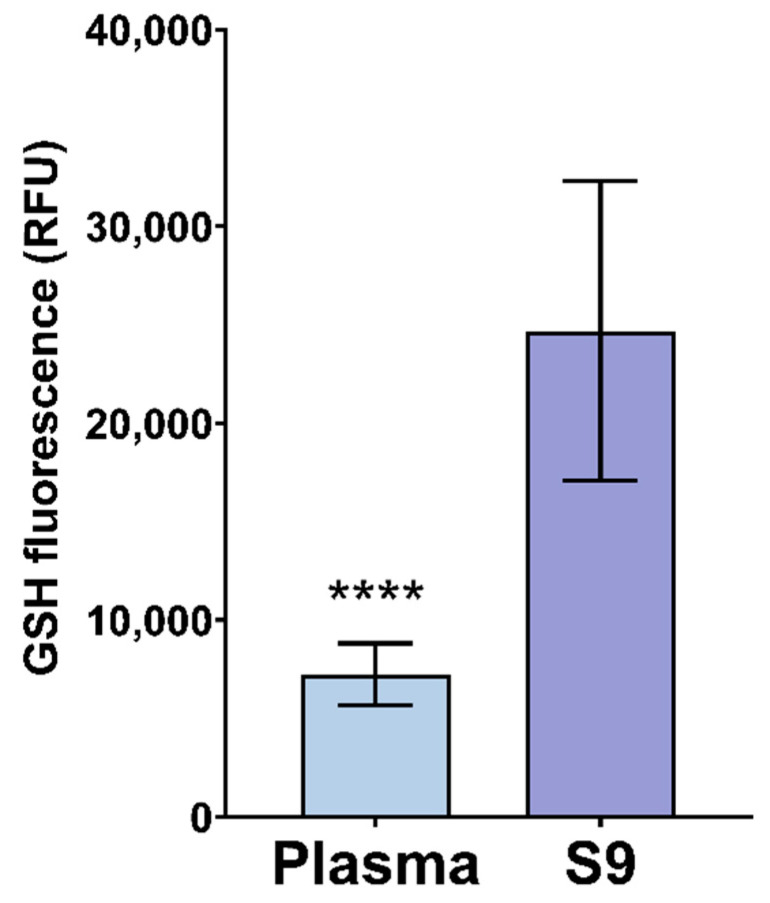
Relative fluorescence (RFU) of reduced glutathione (GSH) in plasma and S9 of white stork (*C. ciconia*) nestlings (*n* = 16), presented as the mean ± SD. Statistical difference is indicated with **** (Welch’s *t*-test, *p* < 0.0001).

The results of fluorescent GSH detection in plasma and S9 for 30 min are shown in Figure 5. Significantly higher GSH fluorescence was reported in S9 than in plasma (*p* < 0.0001). When comparing the variability between responses in these two types of samples, it can be observed that lower variability was observed in plasma compared to S9 (Table 1). Until now, the CellTracker^TM^ Green CMFDA dye for GSH detection was not used in avian blood. However, it was used in zebrafish (*Danio rerio*) embryo and larvae, as well as mouse (*Mus musculus*) embryonic fibroblasts for the purpose of assessing cytotoxicity, apoptosis, and oxidative stress caused by pesticides and silver nanoparticles [41,98,99]. Higher GSH detection was observed in S9, as shown in Figure 5. As S9 contains cellular and subcellular fractions, it was rich with GSH. Most of the GSH is found in the cytoplasm, mitochondria, nucleus, and peroxisomes [100]. Extracellular concentrations of GSH are low [101,102], as shown in Figure 5. In case of smaller sample sizes, usage of plasma for GSH detection is recommended due to the observed lower variability.

CM-H_2_DCFDA dye was used for ROS detection. Different sample concentrations and measurement times were tested, and the final values used were determined based on a linear fluorescence increase and R^2^ ≥ 0.95 (Figure S7). An increase in fluorescence (RFU) was observed in plasma and S9 for 30- and 120-min time periods, respectively. For plasma, an optimal linear increase was observed for 10 min, after which ROS saturation was observed. In the S9 samples, a linear increase was observed for 120 min.

**Figure 6:**
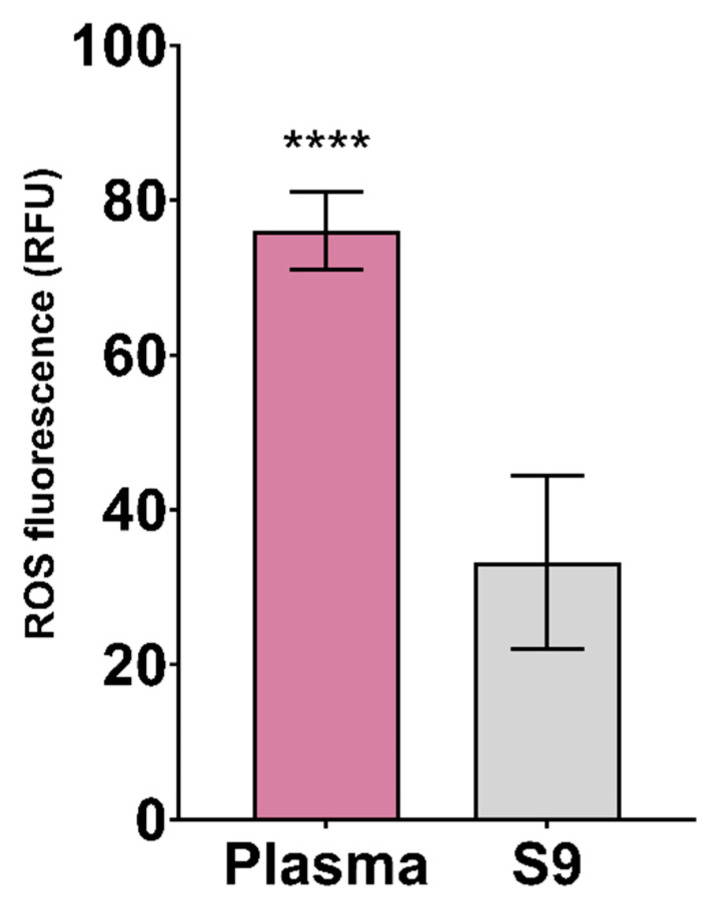
Relative fluorescence (RFU) of the reactive oxygen species (ROS) in plasma and S9 of white stork (*C. ciconia*) nestlings (*n* = 16), presented as the mean ± SD. Statistical difference is indicated with **** (Welch’s *t*-test, *p* < 0.0001).

The results of using a fluorescent dye for measuring ROS detection in plasma and S9 for 10 min are shown in Figure 6. Significantly higher ROS fluorescence was reported in plasma than in S9 (*p* < 0.0001). Lower variability among samples was observed in plasma than S9 (Table 1). Until now, CM-H_2_DCFDA dye was not used in avian blood for ROS detection. However, it was used in zebrafish (*D. rerio*) for the purpose of detecting oxidative stress induced by pesticide exposures [41,99]. Avian erythrocytes have functional mitochondria in terms of ROS production and respiratory activity [103]. Higher ROS detection was observed in plasma compared to S9, as shown in Figure 6. This may be due to extracellular ROS production, induced by external sources (e.g., drugs, pollutants, and radiation) [104]. In case of small sample sizes, using plasma for ROS detection is recommended due to the observed lower variability.

Fluorescence-based oxidative stress detection in blood is a simple, non-destructive method for ROS and GSH detection. Fluorescent detection of GSH and ROS production have never been reported in white stork nestlings. Moreover, to the best of our knowledge, the fluorescent dyes CellTracker^TM^ Green CMFDA and CM-H_2_DCFDA have not been used in avian blood before. However, fluorescent dyes have been successfully used in other model organisms for the purpose of evaluating pesticide exposure and oxidative stress response [41,99]. Fluorescent dyes for GSH and ROS can be used in both plasma and S9 of white stork nestlings for the purpose of evaluating oxidative stress.

## 4. Conclusions

The blood sampling of white stork nestlings is a non-destructive method that can be easily obtained when performed in parallel with ringing. The present study successfully used enzymatic (AChE, CES, GR, and GST) and non-enzymatic (GSH and ROS) biomarkers for determining the basal values in white stork chicks. Fluorescent-based assays, as a novel method for oxidative stress detection in birds, were developed in this study. To get better overall insight into oxidative stress, using enzymatic antioxidants and fluorescence-based oxidative stress detection in two blood fractions will give a better overview of a nestling’s physiological condition. The established protocols can be expanded to other avian species as well. Assessment of the relationship between the biomarkers in the two blood fractions is paramount in order to understand the usefulness of both plasma and S9. This research indicated valuable differences in enzyme activity and oxidative stress detection with fluorescent-based probes for the first time in plasma and S9. Responses for each biomarker in the two blood fractions provide useful information in case of a small sample volume as well as providing overall information about physiological condition. Therefore, we conclude that both plasma and S9 can be used for biomarker analysis.

## Figures and Tables

**Table 1:** Results (sample size (*n*), mean ± SD, and variability) of the enzymatic parameters and fluorescent dyes measured in plasma and S9 of white stork (*C. ciconia*) nestlings.

Parameter	*n*	Plasma			S9		
-	-	Mean	SD	Variability (%)	Mean	SD	Variability (%)
**AChE [nmol min^−1^ mg_prot_^−1^]**	16	14.79	5.12	34.60	3.13	1.26	40.21
**CES [nmol min^−1^ mg_prot_^−1^]**	16	21.53	9.59	44.54	5.85	1.96	33.53
**GST [nmol min^−1^ mg_prot_^−1^]**	16	18.26	7.84	42.93	14.41	2.94	20.37
**GR [pmol min^−1^ mg_prot_^−1^]**	16	98.11	65.67	66.94	840.55	235.42	28.01
**CellTracker^TM^ Green CMFDA (RFU)**	16	7246.07	1571.19	21.68	24683.10	7603.60	30.80
**CM-H_2_DCFDA (RFU)**	16	76.29	5.09	6.68	33.04	11.55	34.94

SD: standard deviation; AChE: acetylcholinesterase; CES: carboxylesterase; GST: glutathione S-transferase; GR: glutathione reductase; CellTracker^TM^ Green CMFDA: dye for glutathione detection; CM-H_2_DCFDA: dye for ROS detection; RFU: relative fluorescence unit.

## Data Availability

All data was included in the manuscript.

## Supplementary Materials

The following are available online at https://www.mdpi.com/article/10.3390/ani11082341/s1, Table S1. DNA concentration; Figure S1. Sex determination results; Figure S2. AChE absorbance; Figure S3. CES absorbance; Figure S4. GST absorbance; Figure S5. GR absorbance; Figure S6. CellTracker^TM^ Green CMFDA dye for GSH detection; Figure S7. CM-H_2_DCFDA dye for ROS detection.
