# Supplementary material for: Application of Non-Destructive Methods: Biomarker Assays in Blood of White Stork (Ciconia ciconia) Nestlings
Source: Animals (Basel). 2021 Aug 8;11(8):2341. doi: 10.3390/ani11082341 (PMC8388685; doi:10.3390/ani11082341)
Supplement: Supplementary file 1 [file animals-11-02341-s001.zip › animals-1335072-supplementary.pdf]

Supplementary information

Application of Non-Destructive Methods: Biomarker Assays in Blood of White Stork (*Ciconia ciconia*) Nestlings

Dora Bjedov[1†], Alma Mikuška[1†], Carina Lackmann[2,3], Lidija Begović[1], Tibor Mikuška[4], Mirna Velki[1*]

[1] Department of Biology, Josip Juraj Strossmayer University of Osijek, , 31000 Osijek, Croatia; dora.bjedov@gmail.com (D.B.); amikuska@biologija.unios.hr (A.M.); lbegovic@biologija.unios.hr (L.B.)
[2] Department of Evolutionary Ecology and Environmental Toxicology, Goethe University Frankfurt,
, 60438 Frankfurt/Main, Germany; lackmann@bio.uni-frankfurt.de
[3] Department of Ecosystem Analysis, Institute for Environmental Research, ABBt–Aachen Biology and Biotechnology, RWTH Aachen University, 52074 Aachen, Germany
[4] Croatian Society for Birds and Nature Protection, 31000 Osijek, Croatia; tibor.kopacki.rit@gmail.com
* Correspondence: mirna.velki@gmail.com or mvelki@biologija.unios.hr; Tel.: +385-(0)31-399-935
† Share first authorship on this work.

Number of pages: 5

Number of tables: 1

Number of figures: 7

Contents:

Table S1. DNA concentration

Figure S1. Sex determination results

Figure S2. AChE absorbance

Figure S3. CES absorbance

Figure S4. GST absorbance

Figure S5. GR absorbance

Figure S6. CellTracker™ Green CMFDA dye for GSH detection

Figure S7. CM-H_2DCFDA dye for ROS detection

Table S1 Quantitative (ng μL^{-1}) and qualitative results of DNA isolation in S9 of white stork (*C. ciconia*) nestlings.

Sample	DNA (ng μL^{-1})	$A_{260/280}$	$A_{260/230}$
**Nestling 1**	2673.80	1.976	2.104
**Nestling 2**	1363.90	1.999	1.703
**Nestling 3**	502.10	1.958	2.130
**Nestling 4**	604.15	2.017	2.005
**Nestling 5**	1118.50	2.028	2.116
**Nestling 6**	1380.30	2.084	2.089
**Nestling 7**	759.10	2.076	2.197
**Nestling 8**	598.65	2.099	2.156
**Nestling 9**	202.50	1.961	2.269
**Nestling 10**	180.00	1.981	1.900
**Nestling 11**	212.85	1.886	2.137
**Nestling 12**	431.60	1.975	2.150
**Nestling 13**	713.00	1.958	1.733
**Nestling 14**	814.00	2.033	1.835
**Nestling 15**	1344.80	1.987	1.913
**Nestling 16**	419.15	1.906	2.133

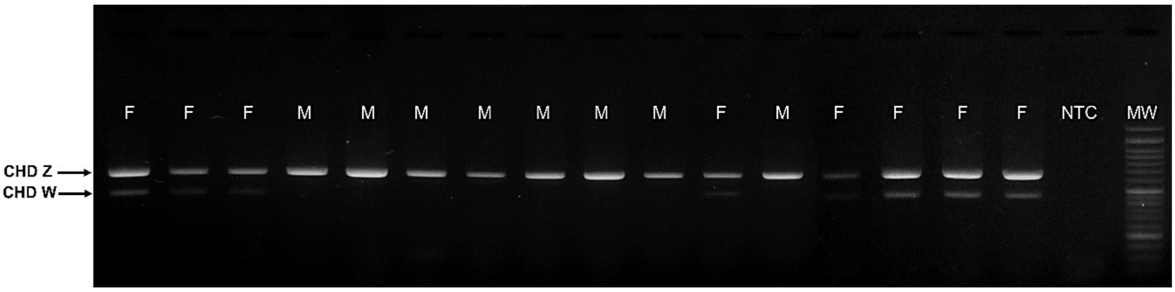

Figure S1 Results of molecular sex-typing. Females (F) have both *CHD Z* and *CHD W* fragments with 600 and 450 bp, respectively. Males (M) have only *CHD Z* fragment, 600 bp. NTC is negative control with no DNA template, and MW is molecular marker.

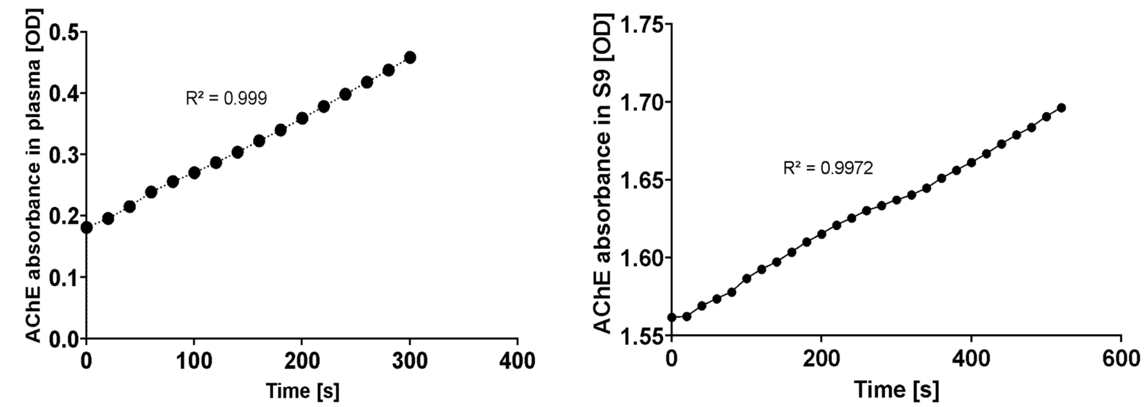

Figure S2 Increase in absorbance (OD) observed during measurement of acetylcholinesterase (AChE) activity in white stork (*C. ciconia*) nestlings. Plasma samples were measured for 5 min and S9 for 10 min (R-squared value of linear trendline is given in the figure).

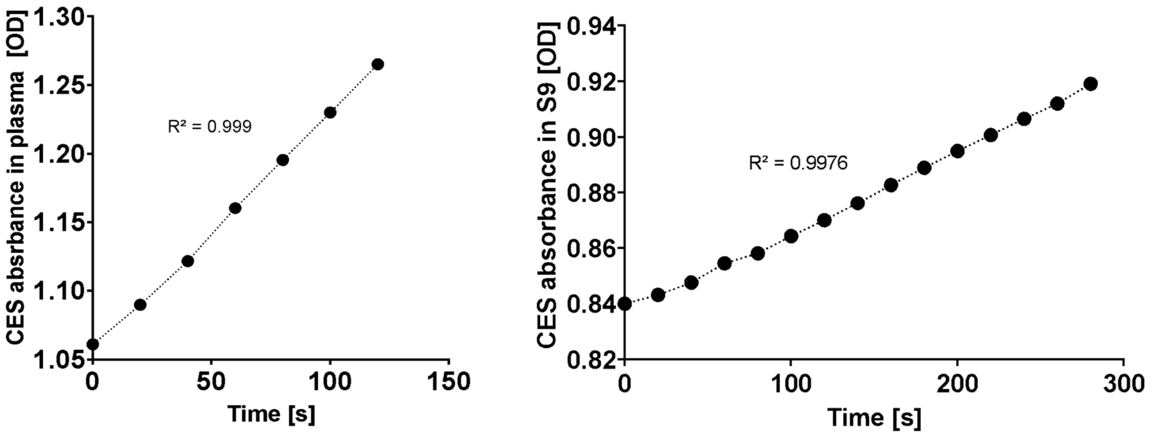

Figure S3 Increase in absorbance (OD) observed during measurement of carboxylesterase (CES) activity in white stork (*C. ciconia*) nestlings. Plasma samples were measured for 2 min and S9 for 5 min (R-squared value of linear trendline is given in the figure).

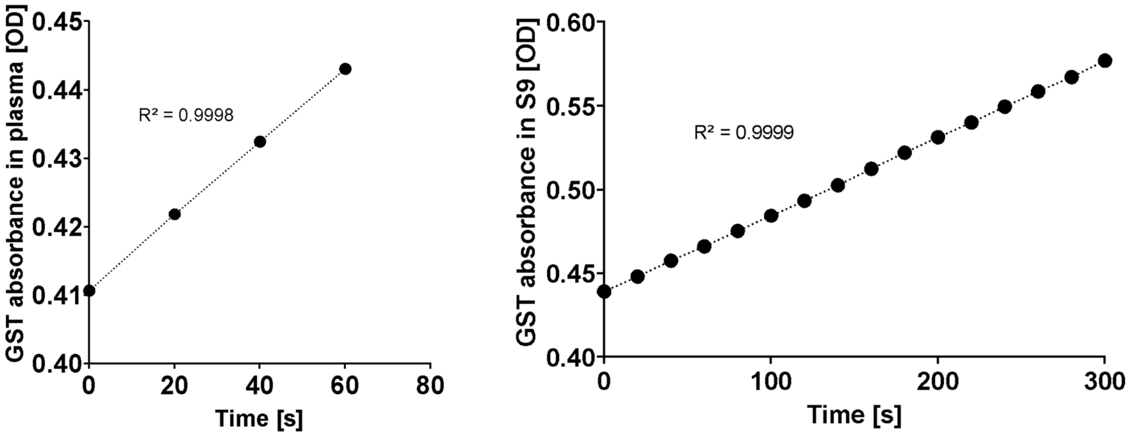

Figure S4 Increase in absorbance (OD) observed during measurement of glutathione S-transferase (GST) activity in white stork (*C. ciconia*) nestlings. Plasma samples were measured for 1 min and S9 for 5 min (R-squared value of linear trendline is given in the figure).

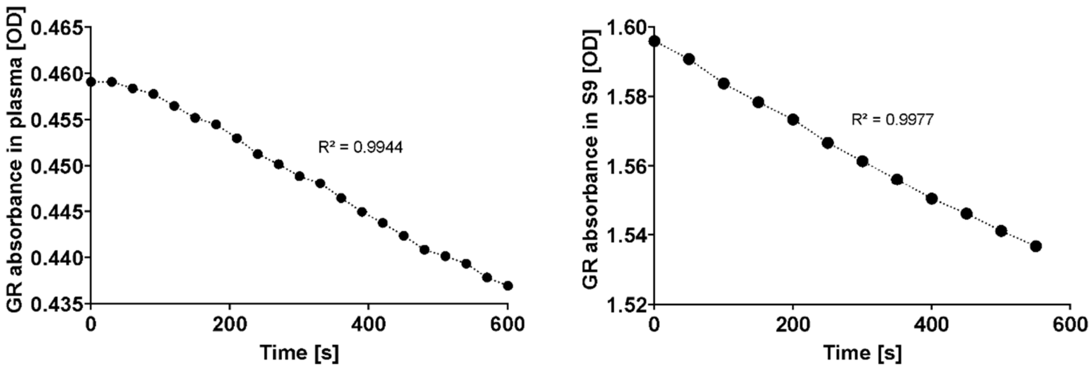

Figure S5 Decrease in absorbance (OD) observed during measurement of glutathione reductase (GR) activity in white stork (*C. ciconia*) nestlings. Plasma and S9 samples were measured for 10 min (R-squared value of linear trendline is given in the figure).

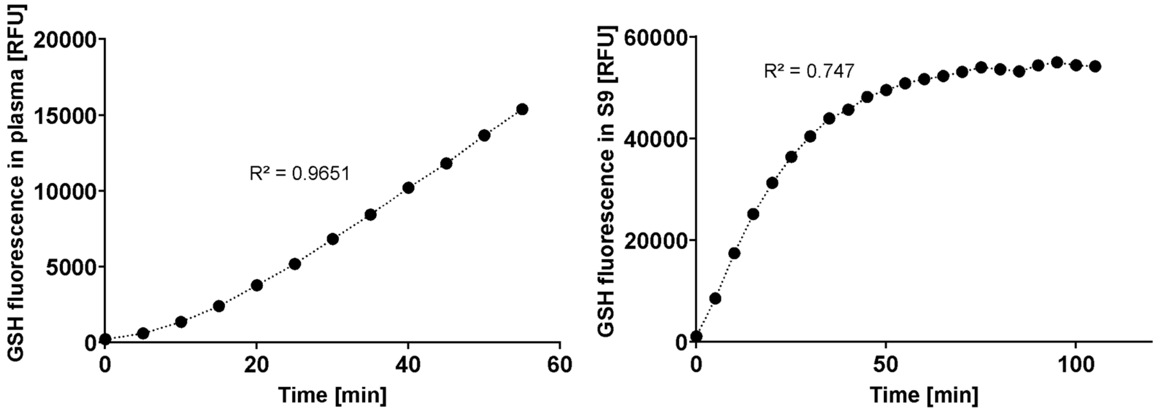

Figure S6 CellTracker™ Green CMFDA dye was used for GSH detection. Increase in fluorescence (RFU) observed during measurement of glutathione (GSH) activity in white stork (*C. ciconia*) nestlings. Plasma samples were measured for 60 min and S9 for 120 min (R-squared value of linear trendline is given in the figure).

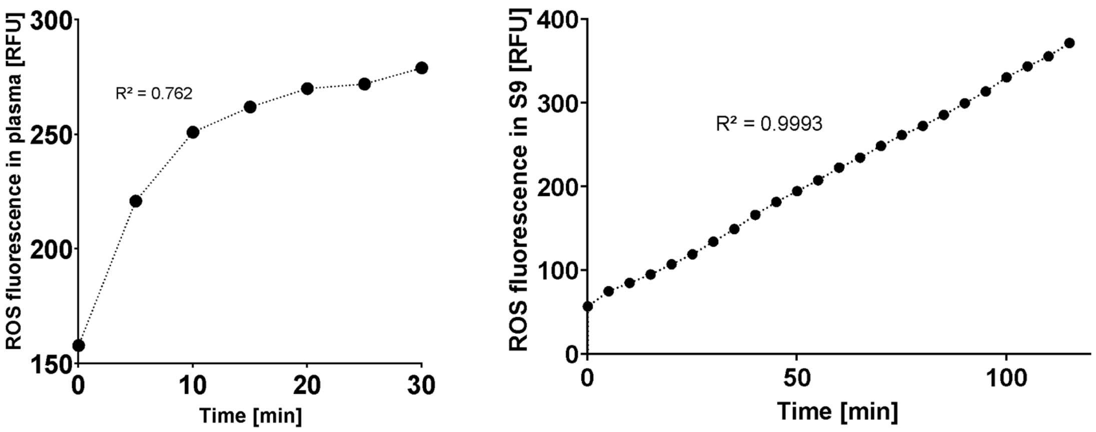

Figure S7 CM-H_2DCFDA dye was used for ROS detection. Increase in fluorescence (RFU) observed during measurement of reactive oxygen species (ROS) activity in white stork (*C. ciconia*) nestlings. Plasma samples were measured for 30 min and S9 for 120 min (R-squared value of linear trendline is given in the figure).
